# Evaluating the diagnostic accuracy of WHO-recommended treatment decision algorithms for childhood tuberculosis using an individual person dataset: a study protocol

**DOI:** 10.1136/bmjopen-2024-094954

**Published:** 2025-09-17

**Authors:** Laura Olbrich, Leyla Larsson, PJ Dodd, Megan Palmer, Minh Huyen Ton Nu Nguyen, Marc d’Elbée, A C Hesseling, Norbert Heinrich, Heather J Zar, Nyanda Elias Ntinginya, Celso Khosa, Marriott Nliwasa, Valsan Verghese, Maryline Bonnet, Eric Wobudeya, Bwendo Nduna, Raoul Moh, Juliet Mwanga, Ayeshatu Mustapha, Guillaume Breton, Jean-Voisin Taguebue, Laurence Borand, Olivier Marcy, Chishala Chabala, James Seddon, Marieke M van der Zalm, Decide-TB Consortium, Chishala Chabala, RaPaed-TB Consortium, Craig Dalgarno, TB-Speed Consortium, Eric Wobudeya, UMOYA Study Group, Margaret van Niekerk

**Affiliations:** 1 Institute of Infectious Diseases and Tropical Medicine, LMU University Hospital, Munchen, Germany; 2 German Center for Infection Research Munich Site, Munchen, Germany; 3 Fraunhofer Institute for Translational Medicine and Pharmacology, Immunology, Infection and Pandemic Research, Munich, Germany; 4 School of Health and Related Research, The University of Sheffield, Sheffield, UK; 5 Department of Paediatrics and Child Health, Faculty of Medicine and Health Sciences, Desmond Tutu TB Centre, Stellenbosch University, Stellenbosch, South Africa; 6 Bordeaux Population Health, University of Bordeaux, Talence, France; 7 Department of Paediatrics and Child Health, Red Cross War Memorial Children’s Hospital, University of Cape Town, Cape Town, South Africa; 8 Unit on Child and Adolescent Health, South African Medical Research Council, Cape Town, South Africa; 9 National Institute of Medical Research—Mbeya Medical Research Centre, Mbeya, United Republic of Tanzania; 10 Instituto Nacional de Saude, Maputo, Mozambique; 11 Helse Nord Tuberculosis Initiative, University of Malawi College of Medicine, Blantyre, Malawi; 12 Department of Child Health, Christian Medical College and Hospital Vellore, Vellore, Tamil Nadu, India; 13 Institut de Recherche pour le Développement (IRD), Université de Montpellier, Montpellier, France; 14 Mulago National Referral Hospital, Kampala, Uganda; 15 Arthur Davidson Children’s Hospital, Ndola, Zambia; 16 Programme PAC-CI, Abidjan, Lagunes, Côte d’Ivoire; 17 Epicentre Mbarara Research Centre, Mbarara, Uganda; 18 Ola During Children Hospital, Freetown, Sierra Leone; 19 Solthis, Paris, France; 20 Chantal Biya International Reference Centre for HIV/AIDS Research on Prevention and Treatment, Yaounde, Cameroon; 21 Clinical Research Group, Epidemiology and Public Health Unit, Institut Pasteur in Cambodia, Phnom Penh, Cambodia; 22 Center for Tuberculosis Research, Division of Infectious Diseases, Johns Hopkins University School of Medicine, Baltimore, MD20600, USA; 23 Department of Paediatrics, University of Zambia, Lusaka, Zambia; 24 Children’s Hospital, University Teaching Hospital, Lusaka, Zambia; 25 Imperial College London, London, UK

**Keywords:** Tuberculosis, Child, Clinical Decision-Making

## Abstract

**Abstract:**

**Introduction:**

In 2022, the WHO conditionally recommended the use of treatment decision algorithms (TDAs) for treatment decision-making in children <10 years with presumptive tuberculosis (TB), aiming to decrease the substantial case detection gap and improve treatment access in high TB-incidence settings. WHO also called for external validation of these TDAs.

**Methods and analysis:**

Within the Decide-TB project (PACT ID: PACTR202407866544155, 23 July 2024), we aim to generate an individual-participant dataset (IPD) from prospective TB diagnostic accuracy cohorts (RaPaed-TB, UMOYA and two cohorts from TB-Speed). Using the IPD, we aim to: (1) assess the diagnostic accuracy of published TDAs using a set of consensus case definitions produced by the National Institute of Health as reference standard (confirmed and unconfirmed vs unlikely TB); (2) evaluate the added value of novel tools (including biomarkers and artificial intelligence-interpreted radiology) in the existing TDAs; (3) generate an artificial population, modelling the target population of children eligible for WHO-endorsed TDAs presenting at primary and secondary healthcare levels and assess the diagnostic accuracy of published TDAs and (4) identify clinical predictors of radiological disease severity in children from the study population of children with presumptive TB.

**Ethics and dissemination:**

This study will externally validate the first data-driven WHO TDAs in a large, well-characterised and diverse paediatric IPD derived from four large paediatric cohorts of children investigated for TB. The study has received ethical clearance for sharing secondary deidentified data from the ethics committees of the parent studies (RaPaed-TB, UMOYA and TB Speed) and as the aims of this study were part of the parent studies’ protocols, a separate approval was not necessary. Study findings will be published in peer-reviewed journals and disseminated at local, regional and international scientific meetings and conferences. This database will serve as a catalyst for the assessment of the inclusion of novel tools and the generation of an artificial population to simulate the impact of novel diagnostic pathways for TB in children at lower levels of healthcare. TDAs have the potential to close the diagnostic gap in childhood TB. Further finetuning of the currently available algorithms will facilitate this and improve access to care.

**STRENGTHS AND LIMITATIONS OF THIS STUDY**

- This individual participant dataset for external validation of the treatment decision algorithms will be derived from well-characterised and comprehensive datasets of children presenting for pulmonary tuberculosis covering a wide range of geographical areas, representing one of the largest datasets of its kind.
- A limitation is that the datasets used to develop the algorithms and this new dataset primarily recruited from tertiary healthcare centres.
- As most children with tuberculosis present primarily at lower levels of healthcare, where healthcare workers with limited training are often responsible for diagnosis, modelling will help to understand the performance of treatment decision algorithms in the target population.
- Finally, a limitation is that the analysis will be a retrospective data-based evaluation; real-life implementation will likely result in different estimates.

## Background

 Tuberculosis (TB) remains a major cause of morbidity and mortality in children worldwide, primarily due to underdiagnosis. The WHO estimates that fewer than half of the children under 15 years with TB, and less than a third of the children under 5 years, are diagnosed.^1^ A modelling study showed that 96% of the children under 15 years of age who died of TB were not diagnosed at the time of death; appropriate and timely treatment could avert some of these deaths.^2^ Efforts aimed at enhancing diagnosis, and consequently increasing access to TB treatment, play a crucial role in diminishing TB morbidity and mortality in children.

**Figure 1:**
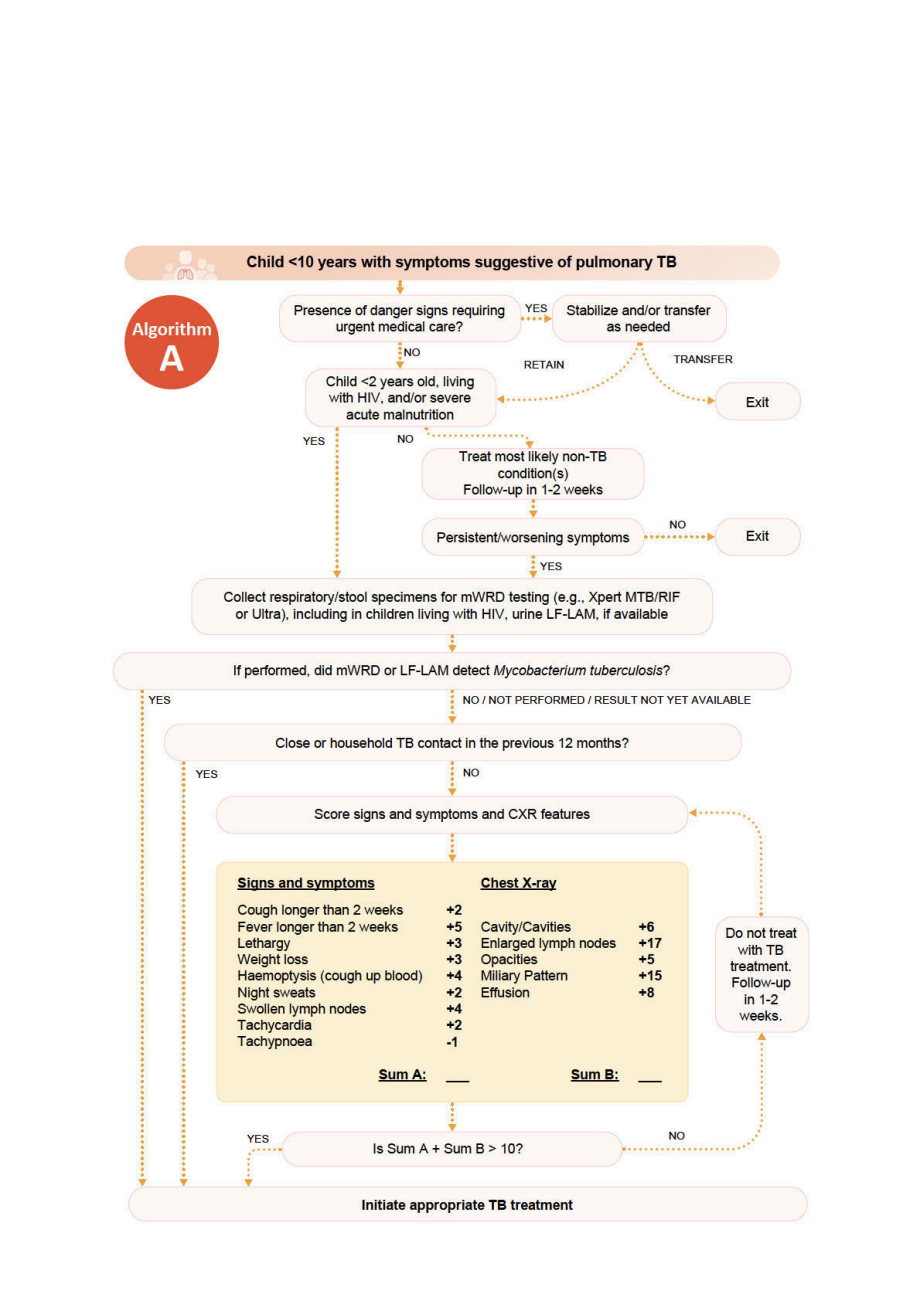
Treatment decision algorithm recommended by the WHO in the 2022 operational handbook for settings with available radiology. MTB, *Mycobacterium tuberculosis; *TB, tuberculosis; LF-LAM, Lateral Flow Urine Lipoarabinomannan Assay; CXR, chest X-ray; mWRD, molecular WHO-recommended rapid diagnostic.

Treatment decision algorithms (TDAs) have been proposed to improve treatment decisions and subsequent TB-related outcomes in children; they guide healthcare workers through a decision tree-based flowchart using information from clinical history, physical examination, laboratory findings and radiology. Of note, TDAs allow for unified decision-making, alleviating the demands on infrastructure and expertise for diagnosing TB disease by providing a standardised tool for treatment decisions. Moreover, TDAs aim to empower healthcare workers at lower levels of care. In 2022, the WHO conditionally recommended the use of TDAs to aid in TB treatment decision-making in children under 10 years with symptoms suggestive of pulmonary TB and suggested two TDAs in the accompanying handbook for use in settings with and without radiography (figure 1).^3^ Both algorithms were designed to maximise sensitivity, resulting in sensitivity estimates of 0.86 (95% CI 0.68 to 0.96) for the algorithm used with radiology and 0.84 (95% CI 0.66 to 0.93) for one without radiology and specificity estimates of 0.37 (95% CI 0.15 to 0.66) and 0.30 (95% CI 0.13 to 0.56), respectively.^4^ The WHO now calls for external validation in light of the 2-year conditional recommendation.^3^ Validating these published TDAs additionally offers a unique opportunity for innovation and improvement; including the inclusion of novel assays, sampling methods and/or novel technologies, which might represent promising strategies to improve the performance of TDAs.

So far, only a few national guidelines have adopted this conditional recommendation, without availability of prospective data, making the validation of these algorithms difficult.^5^ Thus, in this study, we aim to make use of the existing well-characterised paediatric TB cohorts to perform an external validation of the WHO-suggested algorithms as part of the Decide-TB consortium.

### Aims and objectives

Decide-TB (PACT ID: PACTR202407866544155, 23 July 2024) is a multicountry project aiming to assess the effectiveness, feasibility, acceptability, cost and cost-effectiveness, and adoption of a comprehensive TDA-based approach for childhood TB screening/triage, diagnosis and treatment decision-making. Decide-TB has several components/work packages, one of which is a prospective clinical trial that will be implemented under programmatic conditions at district hospital (DH) and primary healthcare (PHC) level in Mozambique and Zambia.

In this study, a work package of the Decide-TB project, we will collate data from child TB diagnostic cohorts to generate an IPD (including RaPaed-TB, UMOYA, TB-Speed HIV, TB-Speed Decentralization (cohort study)). We will then carry out a series of analyses within the work package with the aim of contributing to the external validation of WHO TDAs by evaluating their diagnostic accuracy, as well as that of other published TDAs in detecting paediatric pulmonary TB, and to evaluate the impact of including novel child TB diagnostics in TDAs.

Objectives:

- To externally assess the diagnostic accuracy of published TDAs in detecting paediatric pulmonary TB in a study population of children with presumptive TB.
- To assess the added value of novel tools (including biomarkers and artificial intelligence (AI)-read radiology) and their best placement in existing TDAs and estimate accuracy.
- To evaluate the whole WHO TDA, including the triage step occurring in primary care levels, using modelling on a simulated population.
- To identify clinical characteristics for the prediction of radiological disease severity.

Exploratory objectives include:

- To assess the performance of the WHO TDAs in subgroups of interest, such as high risk versus low risk (defined as in the WHO TDAs) and in different age groups, including adolescents aged 10–14 years.
- To assess whether alternative methods of model choice for the generation of TDAs can achieve meaningful gains in performance, that is, allowing the choice of model to vary by sensitivity and/or specificity threshold.

## Methods/design

### Setting and population

This study includes children evaluated for TB from RaPaed-TB,^6^ UMOYA,^7^ TB-Speed HIV and TB-Speed Decentralization cohort study.^8^,^10^ These studies were rigorously conducted diagnostic accuracy studies that did not contribute to the development of the WHO TDAs. Based on the parent study cohorts, we will generate (1) an IPD from all parent studies (RaPaed-TB, UMOYA, TB-Speed HIV, TB-Speed Decentralization) (‘IPD cohort’); (2) a cohort with results of additional tools available (‘optimisation cohort’) and (3) an artificial cohort generated through modelling and the IPD (‘artificial cohort’).

#### UMOYA

UMOYA is an ongoing prospective observational TB diagnostic cohort study.^7^ The study aims to evaluate novel diagnostic tools and assess the short-term and long-term impact of pulmonary TB and other respiratory diseases on childhood lung health and their quality of life. Children aged <14 years presenting with presumptive TB are recruited from Tygerberg Hospital and Karl Bremer Hospital in South Africa, both regional referral centres.

In total, 600 children were recruited, 500 children with presumptive TB and 100 healthy controls. At enrolment, a minimum of two respiratory samples (gastric aspirate (GA), induced or expectorated sputum) were tested for *Mycobacterium tuberculosis (Mtb*) using smear, culture and Xpert MTB/RIF (Xpert) or Xpert MTB/RIF Ultra (Ultra) (Cepheid, California). In addition, minimally invasive samples were collected for storage and future analysis of novel assays (urine, stool, saliva, blood), and nasopharyngeal aspirates (NPA) were stored for analysis of viral respiratory coinfections. Children were followed up for a total of 6 months, with visits conducted at baseline and at 2, 8, 16 and 24 weeks after enrolment.^7^

#### RaPaed-TB

RaPaed-TB was a prospective diagnostic accuracy cohort study evaluating several novel tests and diagnostic approaches in children <15 years with presumptive pulmonary and/or extrapulmonary TB.^6^ The study recruited 975 children from five sites between January 2019 and June 2021. These centres included tertiary hospitals in South Africa, Malawi, India and urban health facilities in Tanzania and Mozambique. Children were recruited in both inpatient (South Africa, Malawi, India, Mozambique) and outpatient (Malawi, Tanzania, Mozambique) settings.

Two respiratory specimens were collected, on which solid and liquid media cultures (MGIT; Becton Dickinson; New Jersey) were inoculated and one Ultra was performed. Children <5 years underwent NPA for a second Ultra. Additional samples (fingerstick blood, urine and stool) were collected for storage and assessment of novel tests. Children were followed up at months 1 and 3, and month 6 if either still on TB treatment or if they had been unwell at month 3.

#### TB-Speed studies

##### TB-Speed HIV

TB-Speed HIV was a prospective multicentre study evaluating the performance, safety, acceptability, feasibility, cost and cost-effectiveness of the PAANTHER TDA for children living with HIV (CLHIV) with presumptive TB in Côte d’Ivoire, Uganda, Mozambique and Zambia.^11^

Inclusion criteria included children aged >1 month and <14 years with documented and reconfirmed HIV infection and presumptive TB. At enrolment, children were assessed for TB using the PAANTHER TDA; one NPA and one stool were collected and tested with Ultra; in addition, two GA or sputum samples were collected and tested with MGIT and Ultra. Whole blood, plasma, urine and NPA/sputum/GA leftovers collected at enrolment were stored. Once enrolled, children were followed up for 6 months with study visits at day 7, day 15, months 1, 2, 3 and 6. Treatment decisions were largely made using the PAANTHER TDA.

##### TB-Speed decentralization

The TB-Speed Decentralization study aimed to assess the effectiveness, costs and cost-effectiveness of deploying a comprehensive childhood TB diagnosis package on TB case detection at DH and PHC level in six high TB incidence and resource-constrained countries (Cameroon, Cambodia, Côte d’Ivoire, Mozambique, Sierra Leone and Uganda).^8 12^ It also aimed to compare the uptake of different components of the diagnostic package along the cascade of TB care within two specific decentralisation approaches increasing childhood TB case detection. There were two components of the TB-Speed Decentralization study—an operational study and a nested diagnostic cohort study.

Inclusion criteria for the operational research study included sick children (aged <15 years) seeking care at any outpatient department of a DH or PHC centre. The diagnostic package included systematic symptom-based screening of sick children attending the outpatient department, adapted and child-friendly specimen collection methods (ie, NPA and stool or sputum samples), laboratory analysis using Ultra and quality-controlled chest X-ray reading. All children with TB and 10% of children without TB were invited to participate in a prospective diagnostic cohort. All children in this nested diagnostic cohort benefited from CXR (chest X-ray) and had follow-up visits at 2, 4 and 6 months. Children from the nested diagnostic cohort will be included in the IPD, and data from the entire TB-Speed Decentralization study will inform the artificial simulated population for the modelled study component.

### Statistical considerations

#### Variable definitions

Generating clear and well-defined definitions of each variable used in the TDAs is paramount for diagnostic accuracy assessment as individual studies collected information differently. Therefore, a document with version control will be created, which outlines the definitions of variables in each of the TDAs assessed and in each dataset used to generate the IPD. Differences in definitions, depending on the variable, will either be acknowledged as a limitation or will result in change in definitions in other datasets for consistency.

#### Reference standards

Regardless of their initial classification in the respective studies, children will be classified according to the published National Institute of Health (NIH) consensus statements on clinical case definitions for diagnostic TB studies in children.^13^ The clinical case classification will include the following categories: confirmed TB (microbiological confirmation of *Mtb* on respiratory samples via MGIT, LJ culture and/or Ultra), unconfirmed TB (negative microbiological tests on respiratory samples and clinically diagnosed with TB following outlined criteria) and unlikely TB (negative microbiological tests on respiratory samples and not started on TB treatment). In addition, we will explore the use of alternative clinical case definitions in a sensitivity analysis.

**Figure 2:**
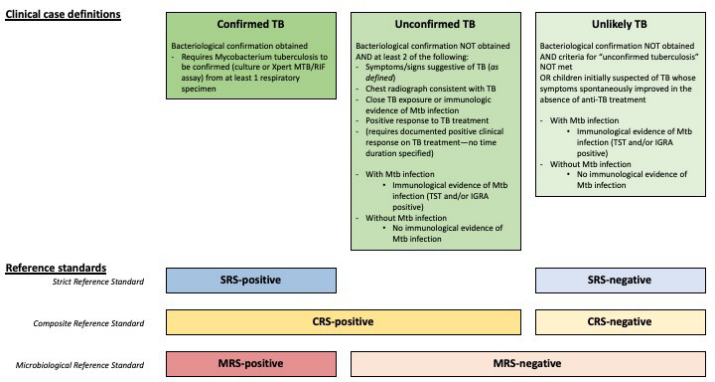
Overview of National Institutes of Health clinical case definitions and reference standards applied in Decide-TB. SRS, strict reference standard; CRS, composite reference standard; MRS, microbiological reference standard; Mtb, *Mycobacterium tuberculosis*; TB, tuberculosis; TST, Tuberculin Skin Test; IGRA, Interferon Gamma Release Assay.

Based on the NIH clinical case definitions, three different reference standards will be applied to estimate diagnostic accuracy of the TDAs with the following degrees of certainty (figure 2):

- A strict reference standard, which solely uses children with a close-to-definite disease status, namely confirmed TB as reference standard positive and unlikely TB as reference standard negative.
- A composite reference standard, which includes children with either confirmed or unconfirmed TB as reference standard positive, and unlikely TB as reference standard negative.
- A microbiological reference standard, which considers solely the microbiological results (culture, Xpert/Ultra) as reference standard positive, with unconfirmed and unlikely TB as reference standard negative.

In addition, we will use a clinician’s decision to treat as an outcome to compare diagnostic accuracy of the TDAs.

#### Analytical components

The work conducted within this study follows the objectives outlined above:

##### Component 1: diagnostic accuracy

In descriptive analyses, we plan to describe all characteristics stratified by country and study. Diagnostic accuracy estimates (including sensitivity, specificity, positive predictive value, negative predictive value) with 95% CIs will be calculated for each TDA using the above-defined reference standards. When computing diagnostic accuracy, while we will do subgroup analyses by study, the overall diagnostic accuracy pooled estimates will be achieved by applying a random-effects meta-analysis. All other modelling-related analyses will include study as a random effect. Receiver operating characteristic curves showing the trade-off between sensitivity and specificity will be plotted, and the area under the curve will be estimated. The analysis will be performed for the entire IPD and then stratified by subgroups of interest.

##### Component 2: improvement of existing TDAs

This workstream will focus on assessing the use of biomarker assays (such as the Xpert host response (MTB-HR) cartridge on blood,^14^ AlereLAM (Abbott, Palatine, Illinois) on urine) and computer-aided CXR reading^15^ within existing TDAs. The purpose is twofold: (1) understand the added value of including such tests in childhood diagnostics as part of the TDA and (2) understand the location within the TDA where they would provide most benefit.

We will generate a subset of the IPD cohort with results of these additional tools available. Children will be randomly selected stratified by age, sex, country, HIV and nutritional status. The Xpert MTB-HR cartridge and urine AlereLAM test will be evaluated on stored samples from each child, and AI-CXR reading will be tested on archived CXR images. This will be done only in children whose parents/caregivers have consented for storage and use of data and samples for future biomarker studies. The added value of these tools will be assessed; score thresholds will be adjusted, and models will be recalibrated to improve the prediction if needed.

##### Component 3: model evaluation in simulated dataset

Most diagnostic accuracy studies in paediatric TB recruit at secondary/tertiary centres, including some of the studies (RaPaed-TB and UMOYA), which are used to generate the IPD. To estimate the performance of the TDAs in a primary care setting, we will generate an in artificial population (produced by means of computer simulation from a generative multivariate statistical model of patient characteristics) of children that would have presented to PHC or DH and were triaged into high-risk and low-risk groups in accordance with the WHO-suggested algorithms. Using data from the IPD, and informed by the TB-Speed Decentralization study, we will model the proportion of children that:

- would have presented to the primary care level or at DH level.
- had symptom resolutions (low-risk patients).
- were triaged into high- and low-risk patients.
- were lost to follow-up prior to repeat evaluations,

This would then allow for an evaluation of the diagnostic accuracy WHO-suggested TDA in settings where it is intended to be used.

##### Component 4: clinical prediction of radiological severe TB disease

A critical challenge in childhood TB is in deciding on treatment duration. Up to two-thirds of children with TB have non-severe TB disease.^16^ Recently, the SHINE trial assessed the efficacy of a shortened TB treatment in children, demonstrating the non-inferiority of 4-month regimens compared with the standard 6-month regimens.^17^ The WHO included the recommendation for this shorter treatment in their latest handbook in 2022.^3^ We will use the SHINE criteria to define radiological disease severity for children in the IPD cohort features.^3 17^ We will then carry out regression analyses to generate a ‘severity-decision algorithm’, using clinical characteristics to predict radiological severity. Among the children with AI-CXR, MTB-HR and urinary AlereLAM evaluated from the optimisation analysis, we will carry out modelling to determine the optimal combination of clinical features and biomarkers to predict radiological severe disease for use in settings where no CXRs are available.

### Missing data

Missing data are expected to occur in two forms: missing data points for variables collected in each parent study, and missing variables from parent studies, which are used in the TDAs. Missing data within the parent studies can occur for various reasons, such as participant non-response, incomplete records or data entry errors. Before merging datasets from different cohorts, a comprehensive assessment of missing data will be conducted, and appropriate strategies will be applied to handle missing data.

### Patient and public involvement

The outputs of the analysis will be discussed with community advisory boards in the settings where the parent studies were performed. In terms of public engagement, we will engage in study dissemination via social media, local dissemination and the Decide-TB website.

### Ethics and dissemination

Each parent study was conducted in line with respective study protocols and with the approval of all local ethical and regulatory bodies (UMOYA N17/08/083, RaPaed-TB 18–205, both TB-Speed HIV and TB-Speed Decentralisation were approved under separate ethical reference numbers^8 12^). All children and/or guardians were consented prior to any study-specific procedures. This study uses retrospective and anonymously shared data and will be set up as a database study. Approval from the relevant ethics committee or institutional review board was sought to ensure compliance with ethical and legal standards; however, as the aims of this study were part of the parent studies’ protocols, a separate approval was not necessary. Human Ethics and Consent to Participate declarations: not applicable as this is a retrospective database analysis.

Findings from this study will be disseminated through peer-reviewed publications and presentations at international scientific conferences. In line with open science principles, curated datasets and accompanying metadata will be deposited in a secure, FAIR-compliant repository, with controlled access for secondary analyses on reasonable request and in accordance with ethical approvals.

## Discussion

TB remains an important cause of morbidity and mortality among children <15 years, particularly those <5 years, largely due to underdiagnosis.^1 18^ Efforts to enhance the sensitivity of diagnostic tools are necessary; it has even been suggested by the WHO and the International Union against Tuberculosis and Lung Disease that treating children based on clinical evidence of TB, even without further diagnostic confirmation, is beneficial, even in the face of potential overtreatment.^3 19^ To address this, TDAs have emerged.^4 20^ Algorithms for treatment initiation aim to facilitate a uniform decision-making process and empower healthcare workers with limited training and/or expertise operating in healthcare settings without the necessary equipment to conduct TB diagnosis. These algorithms utilise all available and easily obtainable evidence and can be applied in decentralised settings and are thus accessible to less-trained healthcare workers. While earlier algorithms were expert driven, those currently under evaluation are the first to be evidence and data driven, developed through analysis of extensive individual person datasets.

External validation of these new diagnostic tools is crucial for their official recommendation by the WHO and their subsequent integration into national TB programmes. However, prospective validation is time-consuming and expensive and can result in a potential lag behind the emergence of new diagnostic pathways. Thus, there is a need for an evaluation method enabling quick and accurate validation, which could prioritise promising methods for further testing. The creation of a new individual person dataset presents a unique opportunity. Not only does it facilitate the external validation of TDAs across different cohorts, but it also allows for algorithm refinement by incorporating novel tests assessed for diagnostic accuracy in parent studies.

Novel assays, sampling strategies and/or novel technologies might represent promising strategies to diagnose children with TB. However, none of these assays or technologies is likely to act as a silver bullet due to the inherent variability in disease presentation and paucibacillary disease among children. Their inclusion and optimal placement within the diagnostic pathway need to be explored and understood. Thus, in addition to the separate evaluation of these novel TDAs and assays, leveraging already collected data and biobanked specimens, together with advanced computational methods, represents an efficient strategy to explore promising diagnostic approaches.

However, a limitation lies in the fact that the datasets used to develop the algorithms and the new validation dataset primarily recruited from tertiary healthcare centres. While, in fact, most children with TB present primarily at lower levels of healthcare, where healthcare workers with limited training are often responsible for diagnosis.^1^ The very young and those with comorbidities such as CLHIV or malnutrition are at high risk of being misdiagnosed and of progressing to severe disease.^1^ To address underdiagnosis in high-incidence settings, TB screening must be decentralised, with tailored solutions for lower levels of healthcare. However, performance in these settings is unclear. The creation of an artificial population from this cohort becomes paramount and facilitates the simulation of the target population. The population entering the score system strongly affects the diagnostic accuracy of the whole TDA and thus ensuring that this is a realistic reflection of the target population is important to understand its potential impact and performance. These artificial populations can help to simulate how interventions perform in different populations of interest or investigate alternative scenarios and support decision-making.^21^

In conclusion, this study will externally validate the WHO TDAs in a large, well-characterised and diverse IPD derived from four paediatric presumptive TB cohorts. This database will serve as a springboard for the assessment of the inclusion of novel tools and the generation of an artificial population to simulate the impact of novel diagnostic pathways in lower levels of healthcare. TDAs have the potential to close the diagnostic gap in childhood TB, and further finetuning of the currently available algorithms will facilitate this.
