# Supplementary material for: Evaluating the diagnostic accuracy of WHO-recommended treatment decision algorithms for childhood tuberculosis using an individual person dataset: a study protocol
Source: BMJ Open. 2025 Sep 17;15(9):e094954. doi: 10.1136/bmjopen-2024-094954 (PMC12458782; doi:10.1136/bmjopen-2024-094954)
Supplement: Supplementary file 1 [file bmjopen-2024-094954.reviewer_comments.pdf]

PEER REVIEW HISTORY

BMJ Open publishes all reviews undertaken for accepted manuscripts. Reviewers are asked to complete a checklist review form and are provided with free text boxes to elaborate on their assessment. These free text comments are reproduced below.

ARTICLE DETAILS

Title (Provisional)

Evaluating the diagnostic accuracy of WHO-recommended treatment decision algorithms for childhood tuberculosis using an individual person dataset - a study protocol

Authors

Olbrich, Laura; Larsson, Leyla; Dodd, PJ; Palmer, Megan; Nguyen, Minh Huyen Ton Nu; d'Elbée, Marc; Hesseling, A C; Heinrich, Norbert; Zar, Heather J; Ntinginya, Nyanda Elias; Khosa, Celso; Nliwasa, Marriott; Verghese, Valsan; Bonnet, Maryline; Wobudeya, Eric; Nduna, Bwendo; Moh, Raoul; Mwanga, Juliet; Mustapha, Ayeshatu; Breton, Guillaume; Taguebue, Jean-Voisin; Borand, Laurence; Marcy, Olivier; Chabala, Chishala; Seddon, James; van der Zalm, Marieke M; Consortium, TB-Speed; Study Group, UMOYA; Consortium, RaPaed-TB; Consortium, Decide-TB

VERSION 1 - REVIEW

**Reviewer**	**1**
**Name**	**Majidulla, Arman**
**Affiliation**	**JHU, International Health**
**Date**	**03-Jan-2025**
**COI**	**None**

Tuberculosis is a disease of poverty, and as such the use of effective TDAs in many contexts are vital for the initiation of lifesaving treatment for children and adolescents. Their importance is furthered due to the fact that the availability of diagnostic testing in some poor urban/peri-urban/rural areas can be limited and inconsistent. This is a timely and important study which seeks to evaluate the WHO TDA in an important population using person level data.

While the available data covers a variety of different contexts, and high burden settings such as India and South Africa, the authors are themselves limited by the limitations of the respective studies from which they seek to form an IPD. Under diagnosis is a function of the setting within which diagnosis is first sought, and may be heterogeneous and temporally

inconsistent. Given the diverse grouping of research projects chosen to develop the IPD, this is not a ubiquitous concern but may be a limitation for some.

The authors acknowledge this concern to a certain extent, though as this proposed study materializes it would be valuable to provide some insight into how TDAs are differentially interpreted and applied in different contexts, including any insights into whether these differential interpretations are a cause for concern.

**Reviewer**	**2**
**Name**	**Gebregergs, Gebremedhin Berhe**
**Affiliation**	**Mekelle University College of Health Sciences**
**Date**	**12-Mar-2025**
**COI**	**None**

Title: Evaluating the diagnostic accuracy of WHO-recommended treatment decision algorithms for childhood tuberculosis using an individual person dataset - a study protocol

Introduction

1. In the introduction, the author should describe the setting in which WHO's TDAs are recommended to be applied.
2. Authors have described that few national guidelines have adopted TDA. What impact did algorithm have on case detection and child mortality due to TB in these nations?

Method

3. There is a high level of heterogeneity in the population and setting. For example, there are differences between inpatient and outpatient care, HIV-positive and mixed patients, and referral hospitals and primary healthcare units. However, a plan for handling this variability is not described.

VERSION 1 - AUTHOR RESPONSE

Reviewer: 1
Tuberculosis is a disease of poverty, and as such the use of effective TDAs in many contexts are vital for the initiation of lifesaving treatment for children adolescents. Their
diagnostic testing in some poor urban/peri-urban/rural areas can be limited and inconsistent. This is a timely and important study which seeks to evaluate the WHO TDA in an important population using
person level data.

While the available data covers a variety of different contexts, and high burden settings such as India and South Africa, the authors are themselves limited by limitations of the respective studies from which they seek to form an IPD. Under diagnosis is a function of the setting within which diagnosis is first sought, and may be heterogeneous and temporally inconsistent. Given the diverse grouping of research projects chosen to develop the IPD, this is not a ubiquitous concern but may be a limitation for some.

The authors acknowledge this concern to a certain extent, though as this proposed study materializes it would be valuable to provide some insight into how TDAs are differentially interpreted and applied in different contexts, including any insights into whether these differential interpretations are a cause for concern.

Reply: We appreciate the feedback of the reviewer. The IPD analysis is indeed hampered by the retrospective nature of the study, which is highlighted in the limitations of the study. The heterogeneity of the settings in which the TDAs can be used indeed will have an impact on outcomes of the study. Where possible, we will stratify our analysis by site-level characteristics such as healthcare setting (e.g., primary vs. tertiary care) to get better insights into the use of the TDAs in different settings. Please see added text on this page 6, lines 161-167.

Reviewer: 2
Mr. Gebremedhin Berhe Gebregergs, Mekelle University College of Health Sciences
Comments to the Author: ***Please see attached file for any additional comments***
Title: Evaluating the diagnostic accuracy of WHO-recommended treatment decision algorithms for childhood tuberculosis using an individual person dataset—a study protocol

Reply: We appreciate the constructive feedback of the reviewer.

Introduction

1. In the introduction, the author should describe the setting in which WHO's TDAs are recommended to be applied.
   Reply: The TDAs are mostly focussed on settings where no paediatric TB expertise is available, generally in lower levels of care. We have clarified this in page 1 lines 14.
2. The authors have described that few national guidelines have adopted TDA. What impact did the algorithm have on case detection and child mortality due to TB in these nations?
   Reply: To the best of our knowledge, there is currently no data available yet on the prospective evaluation of the TDAs and how this has impacted the case detection and child mortality due to TB. As part of the larger Decide-TB project this is currently prospectively evaluated in Zambia and Mozambique. We have added this in page 1 lines 25 -26.

Method

3. There is a high level of heterogeneity in the population and setting. For example, there are differences between inpatient and outpatient care, HIV-positive and mixed patients, and referral hospitals and primary healthcare units. However, a plan for handling this variability is not described.

Reply: We thank the reviewer for this comment and whilst the heterogeneity of this study is a strength as it is more representative of different child populations, we agree that this must be carefully considered in analyses. In descriptive analyses, we plan to describe all characteristics stratified by country. When computing diagnostic accuracy, whilst we will do subgroup analyses by study, the overall diagnostic accuracy pooled estimates will be achieved by applying a random-effects meta-analysis. All other modelling-related analyses will include study as a random effect. We have added some clarification on this in the text, see page 6, lines 161-167.

.

Reviewer: 1

If you have selected ‘Yes’ above, please provide details of any competing interests.: Not applicable

Reviewer: 2

If you have selected ‘Yes’ above, please provide details of any competing interests.: I have no conflict of interest
